# P075. Secondary headache due to bilateral dissection of vertebral arteries

**DOI:** 10.1186/1129-2377-16-S1-A129

**Published:** 2015-09-28

**Authors:** Valeria Villella, Ilenia Molinaro, Guido Granata, Annacarla Finucci, Massimo Granata

**Affiliations:** U.O.C. Immunologia Clinica A, Dipartimento di Medicina Clinica, Policlinico Umberto I, Sapienza Università di Roma, Rome, Italy

## Intoduction

Headache with or without neck pain can be the only manifestation of cervical arterial dissection. Headache is the most common and the most frequent onset symptom in cervical arterial dissection. Section 6.5.1 of the International Classification of Headache describes facial or neck pain imputable to arterial dissection. The described diagnostic criteria are: acute onset of facial or neck pain with or without other neurological signs or symptoms, developing in close temporal relationship and on the same side of the dissection and disappearing within 1 month. In addition, the dissection must be diagnosed by appropriate vascular/neuroimaging investigations. Post-traumatic and spontaneous dissection occurrence is described in the literature; these occurrences although rare should be considered and promptly investigated in order to begin an effective treatment.

## Case presentation

A previously healthy 37-year-old nurse was admitted in our department for acute onset of headache with vertigo and nausea. In her past medical history there were post-partum onset of major depression and anorexia nervosa. At the Emergency Department she underwent a brain-CT and lumbar puncture which showed no significant alterations. Once she was transferred to our department we required an MR of the brain that showed no pathological signs. We considered the diagnosis of tension-type headache and she was given, steroids and benzodiazepine treatment. Two days after starting the therapy no improvement of the pain was observed. She reported persistence of headache worsening during orthostatic position; neurological examination revealed nistagmoid movements at the higher quadrants. The worsening headache with bilateral continuous occipital and posterior cephalagia, associated with nausea and persisting after two weeks from the established therapy led us to perform a TSA echo-color-doppler followed by angio-TC. These exams showed alterations suspected for vertebral dissection, and a diagnostic angiography confirmed bilateral dissection of vertebral arteries.

## Discussion

Headache and migraine are the presenting symptoms in 57-92% of the carotid artery dissections and in 69-72% of vertebral artery dissections. Pain localization is not specific for the dissected artery; However, carotid dissections tend to present with frontal pain, while pain resulting from the dissection of the vertebral artery is easier to posterior or occipital. Pain localized in the eye, ear or face more easily indicates involvement of the carotid artery. Cervical artery dissection occurs when the tonaca intima is damaged due to direct trauma or an anomaly. Thus, blood can fill the space created between the layers of the artery wall forming clots, potential cause of stroke, pseudo aneurysms and occlusions of vessels. Intracranial dissections can cause a subarachnoid hemorrhage. Considering that cervical artery dissections may present with common signs or symptoms such as headaches, neck pain, neurological deficits and stroke, it is important to consider and rule out the possibility of a dissection. CT angiography, angio-MR and digital subtraction angiography are useful for diagnosis. The first-line therapy for the treatment of spontaneous or traumatic dissection is an anticoagulant or antiplatelet therapy to reduce the risk of stroke. There may be an indication for endovascular treatment or surgery. There is a significant risk of recurrence or re-bleeding that must be taken into account. In particular, the recurrence occurs in 10-28% of cervical artery dissections with the higher risk during the first two months from the first dissection. Patients with both cervical arteries involved (such as the reported case) have a four times higher risk of recurrent dissection. Cases presenting with triple or quadruple dissections are rare, respectively 1.5% and 0.1% of spontaneous dissections. They are found more easily in women and the risk of relapse is the same as when two arteries are involved. The risk of recurrent dissection increases in patients with a positive family history and in young patients (<45 years). If there is a positive family history for the disease, prospective studies have shown a recurrence rate from 50% to 100%, versus 6-22% in patients with negative family history for arterious dissection.

Written informed consent to publication was obtained from the patient(s).

